# The impact of high progesterone levels on the day of HCG administration in assisted human reproduction treatments

**DOI:** 10.5935/1518-0557.20180020

**Published:** 2018

**Authors:** Michelli S Tanada, Ivan H Yoshida, Monise Santos, Caroline Z Berton, Elen Souto, Waldemar P de Carvalho, Emerson B Cordts, Caio P Barbosa

**Affiliations:** 1 Instituto Ideia Fértil de Saúde Reprodutiva, Santo André - SP, Brazil; 2 Faculdade de Medicina do ABC, Santo André - SP, Brazil

**Keywords:** progesterone, top quality D3, assisted human reproduction

## Abstract

**Objective:**

Progesterone is a steroid hormone that acts on the endometrium. It is known for producing physical and mood-related side effects. Few studies have looked into how progesterone levels affect embryo development and quality. This study aimed to find a cutoff level for serum progesterone on the day of HCG administration from which embryo quality is impaired.

**Methods:**

The study included 145 cycles, from which 885 oocytes and 613 embryos were obtained. All patients had their serum progesterone levels measured on the day of HCG administration. Data sets were collected from patient medical records. The chi-square test was used to assess qualitative variables and the Mann-Whitney test to evaluate quantitative variables.

**Results:**

Statistical analysis revealed that serum progesterone levels and reproductive variables were not significantly associated. In regards to oocyte maturity, however, when progesterone levels were greater than 1.3 ng/mL the probability of oocytes being immature increased by 12.7%. The fragmentation rate of embryos categorized as "top quality" in D3 increased proportionately to increases in progesterone levels (12.23%).

**Conclusion:**

High progesterone levels appeared to be correlated with increased embryo fragmentation rates, but high serum levels of the hormone on the day of HCG administration had no impact on reproductive variables and were not associated with impaired embryo development.

## INTRODUCTION

Progesterone is a steroid hormone synthesized in the cells of the corpus luteum in the ovaries. It is involved in a number of functions such as endometrial gland formation and secretion, cervical mucus secretion, preparation and maintenance of pregnancy, and breast development, among others.

For decades, most scientific studies have reported that high levels of progesterone on the day of HCG administration were related to impaired endometrial receptivity and decreased pregnancy rates in fresh cycles (Huang *et al*., 2016). Some authors reported increased progesterone levels on the day of HCG administration in up to 38% of assisted reproductive technology (ART) cycles (Bosch *et al*., 2003).

A receptive endometrium relies on the interaction between estrogen and progesterone. Endometrial receptivity is driven by exposure to progesterone following sufficient exposure to estrogen. Progesterone levels increase during the late follicular phase of ovarian stimulation for in vitro fertilization (IVF), while serum progesterone levels rise as the diameter of the follicles increases. In ovarian stimulation, each of the many growing follicles contributes to increase progesterone levels in systemic circulation (Lawrenz & Fatemi, 2017). Endometrial receptivity is triggered after exposure to progesterone and estrogen, thus the time for which the endometrium is receptive and able to tolerate interactions is limited.

In ART cycles, when progesterone levels are high the embryos are vitrified so that the endometrium is subsequently prepared. Factors such as embryo morphology also affect pregnancy rates. Although the reports in the literature are conflicting, high levels of progesterone on the day of HCG administration are believed to produce negative effects on oocyte and embryo quality.

The first investigations into the adverse effects of high levels of progesterone on the day of HCG administration on embryo quality were performed in 1993 and 1994. No statistically significant association was found between high levels of progesterone on the day of HCG administration and embryo quality (Hofmann *et al*., 1993; Silverberg *et al*., 1994). Other authors have looked into a possible association between increased progesterone levels and good quality cleavage stage embryos (Huang *et al*., 2016). Although statistically significant differences were not found, the authors noted that embryo quality was significantly reduced when serum levels were greater than 2.0 ng/mL.

In view of the impact of progesterone on ART and the controversy looming over the small number of papers produced on the topic, this study aimed to find the serum progesterone levels on the day of HCG administration from which embryo quality is impaired.

## MATERIAL AND METHODS

### Case series

This study included 145 ART cycles performed from December of 2016 to May of 2017. These cycles yielded 885 oocytes and 613 embryos. Serum progesterone levels of all patients were measured on the day of HCG administration.

Exclusion criteria: patients aged 37 years or older; absence of oocytes on the day of follicular aspiration; cycles with indication for PGD/PGS; and patients diagnosed with ovarian hyperstimulation syndrome (OHSS).

### Serum progesterone level measurement

Serum progesterone levels were measured on the day of HCG administration with a COBAS E411 analyzer using the Progesterone II reagent made by Roche (Germany).

### Reproductive variables

The following reproductive variables were studied for possible correlations with progesterone values:

- Oocyte maturation
- Fertilization rate
- Top quality embryos in D3
- Blastocyst formation

### Statistical analysis

Qualitative variables were described using absolute and relative frequencies. The quantitative variables did not follow a normal distribution, and were therefore described with medians and percentiles (25% and 75%). The chi-square test was applied to analyze the association between qualitative variables. The Mann-Whitney test was used to analyze the differences of the quantitative variables in relation to the cutoff value. *p*-values <0.05 were considered statistically significant.

## RESULTS

Serum progesterone levels were analyzed vis-à-vis the following reproductive variables: oocyte maturation, fertilization rate, top quality embryos in D3, and blastocyst formation.

Statistical analysis found that when serum progesterone levels were greater than 1.3 ng/mL, 12.7% of the oocytes were immature, versus 9% when serum progesterone was below the cutoff value (*p*=0.333).

Increased serum progesterone levels occurred concurrently with poorer cleavage stage embryo quality. When progesterone levels were greater than 1.3 ng/mL, 12.23% of the embryos had higher fragmentation rates than the embryos with progesterone levels below the cutoff value (*P* = 0.331).

Fertilization and blastulation rates were unaffected by serum progesterone levels.

## DISCUSSION

Several authors have discussed the negative impacts of high serum progesterone levels on the endometrium. However, the effects of progesterone on oocytes and embryos have been scarcely studied. No statistically significant correlations were found between serum progesterone levels and oocyte maturity, fertilization rate, top quality embryos in D3, and blastocyst formation in this study. However, a tendency toward negative impact on oocyte maturity and top quality embryos in D3 was observed.

A study looked into 4,236 cycles to assess the impact of serum progesterone levels on embryo quality. The authors found no negative association between elevated levels of progesterone and cleavage stage embryo quality, but noted that serum progesterone levels greater than 2.0 ng/mL were significantly associated with lower numbers of top quality embryos in D3 (Huang *et al*., 2016). The same authors (Huang *et al*., 2014) conducted a comparative study with 9,858 cycles considering the clinical characteristics and outcomes of cycles of patients submitted to IVF and late ICSI and found that high levels of progesterone on the day of HCG administration might have adversely affected oocyte fertilization, mainly when serum levels were greater than 1.5 ng/mL.

In the present study, elevated levels of progesterone did not significantly affect fertilization rates. A retrospective study carried out from 2013 to 2016 including 1415 cycles assessed whether elevated serum progesterone levels on the day of HCG administration were associated with decreased formation rates of good quality blastocysts. The authors reported an association between higher levels of progesterone and low formation rates of good quality blastocysts (Vanni *et al*., 2017).

In this study, high levels of progesterone did not significantly affect blastulation rates and embryo quality on the day of HCG administration. Other authors reported that high progesterone levels on the day of HCG administration were more frequently observed in women with recurring IVF failure (Liu *et al*., 2013).

The reasons underlying the elevation of serum progesterone levels during ovarian stimulation remain unclear. However, recent publications indicated that the follicle stimulating hormone (FSH) surge at the end of the follicular phase might be the main cause of elevation of progesterone.

## CONCLUSION

This study indicated that increased serum progesterone levels on the day of HCG administration might adversely affect oocyte maturation and the formation of top quality embryos in D3. Further studies with larger samples might help increase the accuracy of reproductive variable assessment.
